# Expression, purification and structural analysis of the *Pyrococcus abyssi *RNA binding protein PAB1135

**DOI:** 10.1186/1756-0500-3-97

**Published:** 2010-04-09

**Authors:** Juliana S Luz, João ARG Barbosa, Celso RR Ramos, Carla C Oliveira

**Affiliations:** 1 Department of Biochemistry, Institute of Chemistry, University of São Paulo, 05508-000, São Paulo, SP, Brazil; 2 Center for Structural Molecular Biology, Brazilian Synchrotron Light Laboratory, LNLS, 13083-970, Campinas, SP, Brazil; 3 Current address: Pós-Graduação em Ciências Genômicas e Biotecnologia, Universidade Católica de Brasília, 70790-160, Brasília, DF, Brazil; 4 Current address: Department of Helminthology, Fiocruz, 21045-900, Rio de Janeiro, RJ, Brazil

## Abstract

**Background:**

The gene coding for the uncharacterized protein PAB1135 in the archaeon *Pyrococcus abyssi *is in the same operon as the ribonuclease P (RNase P) subunit Rpp30.

**Findings:**

Here we report the expression, purification and structural analysis of PAB1135. We analyzed the interaction of PAB1135 with RNA and show that it binds efficiently double-stranded RNAs in a non-sequence specific manner. We also performed molecular modeling of the PAB1135 structure using the crystal structure of the protein Af2318 from *Archaeoglobus fulgidus *(2OGK) as the template.

**Conclusions:**

Comparison of this model has lead to the identification of a region in PAB1135 that could be involved in recognizing double-stranded RNA.

## Background

Despite the recent progress in various genome analysis projects, about a quarter of the archaeal genomes encode functionally uncharacterized proteins, which are almost all only common to other archaeal species [1-4]. *Pyrococcus abyssi *PAB1135 protein function has not yet been characterized, and it is classified in the family domain of unknown function 54 (DUF54) and in the uncharacterized protein family 0201 (UPF0201). This group's members have been annotated as conserved hypothetical proteins in 46 archaeal species. Some of these proteins were annotated as possible exosome subunits (TK1451 - GI: 57641386, *Thermococcus kodakarensis*; MK0388 - GI: 20093826, *Methanopyrus kandleri; *Msp1244 - GI: 84490032, *Methanosphaera stadtmanae*) [4-6], but analysis of completely sequenced *Pyrococcus abyssi *genome revealed the presence ofPAB1135 gene in the same operon as Pa1136, the ribonuclease P (RNase P) subunit Rpp30 [7].

RNase P is an endoribonuclease responsible for maturation of tRNAs in all domains of life. RNase P is a ribonucleoprotein (RNP) complex, formed by one RNA molecule and a variable number of protein subunits, depending on the organism. Bacterial RNase P contains one protein, whereas the archaeal relative contains at least four proteins, and in humans, it contains at least 10 protein subunits [8,9]. *Pyrococcus horikoshii *RNase P has been shown to be formed by one catalytic RNA and the proteins Ph1481, Ph1601, Ph1771, and Ph1877, which show homology to the human RNase P subunits hPop5, Rpp21, Rpp29, and Rpp30, respectively [10]. The structures of these *P. horikoshii *proteins have been solved and a possible arrangement of the protein complex has been proposed [11].

Although the function of *Pyrococcus abyssi *PAB1135 has not been characterized, nor its association with RNase P complex, the genome location of the gene suggests that PAB1135 is involved in RNA metabolism. Here we show that PAB1135 binds RNA *in vitro*, showing higher affinity for double-stranded RNAs. In addition, structural analysis of PAB1135 by molecular modeling indicates a possible region for protein-RNA interaction.

## Methods

### Cloning of PAB1135 sequence

The *Escherichia coli *strains used in this study were DH5α and BL21-CodonPlus (DE3)-RIL (Stratagene). Plasmid DNA was extracted using Qiagen plasmid purification systems. Restriction enzymes and other DNA-modifying enzymes were used as recommended by the manufacturer (New England Biolabs). PAB1135 coding sequence was PCR-amplified from *P. abyssi *GE5 genomic DNA Genomic DNA (kindly provided by Dr. Patrick Forterre from Institut de Génétique et Microbiologie, Université Paris Sud, France) using primers PAB1135for (5'-GTTAGGGG*GGATCC***ATG**GCAG-3') and PAB1135rev (5'-CGG*CCTCGA*GTC**AAT**CCTCCC-3'). The restriction sites used are underlined in the primers' sequences, and the start and stop codons are in bold. A DNA fragment of 462 bp was obtained from the PCR reaction and inserted into vector pET28a (Novagen), digested with *BamH*I-*Xho*I. A 21 kDa tagged protein His-PAB1135 was produced from this plasmid

### Expression and purification of the recombinant protein

The pET28a-PAB1135 was transformed into the *E. coli *BL21-CodonPlus (DE3)-RIL strain. The transformed cells were grown at 37°C in 2xTY medium supplemented with 20 mg/L kanamycin and 17 mg/L chloramphenicol. The expression of His-PAB1135 was induced for two hours with 0.5 mM IPTG. Cells were harvested by centrifugation, suspended in buffer A (30 mM Tris-HCl, pH 8.0, 500 mM NaCl, 5 mM imidazole) and lysed in a French press. The lysate was heated at 85°C for 30 min and cooled on ice for 15 min. After centrifugation at 20,000 × *g *for 30 min, the supernatant was fractionated by affinity chromatography in Ni-NTA-agarose (Qiagen). The purified fractions were analyzed by SDS-PAGE.

### Thrombin and Trypsin digestion and analysis

To remove the His-tag from PAB1135, tagged protein was incubated with thrombin for 8 h at room temperature, as recommended by the manufacturer (GE Healthcare). Trypsin treatment was performed by incubating His-PAB1135 with 0.1% trypsin solution (0.1 mg/ml) for 2 hours at room temperature, followed by the addition of 1 mM PMSF. His-PAB1135 and its tryptic cleavage products were subjected to SDS-PAGE and transferred to PVDF membranes (BioRad), which were incubated with an anti-poly-histidine antibody (GE Healthcare). The immunoblots were developed using the ECL system (GE Healthcare).

### Gel filtration and circular dichroism

Gel filtration assays of trypsin-treated PAB1135 were carried out in a superdex 75 XK 16/60 column (GE Healthcare) in the presence of 50 mM Tris-HCl pH 8.0, 150 mM NaCl, 0.5 mM EDTA. Apparent molecular masses were assessed based on the retention time of the molecular mass markers (low molecular mass gel filtration calibration kits, GE Healthcare: bovine serum albumin, 67 kDa; ovalbumin, 43 kDa; chymotrypsinogen, 25 kDa; ribonuclease A, 13.7 kDa).

For the circular dichroism analysis, PAB1135 was dialyzed against buffer B (50 mM Tris-HCl pH 8.0, 200 mM NaCl, 5 mM MgCl_2_, 1% glycerol, 0.02% tween 20, 1 mM EDTA) and concentrated to 2 mg/ml using Amicom ultra (Millipore). The circular dichroism experiments were conducted on a JASCO 810 spectropolarimeter using a 1 mm path length cuvette. The K2d program [12] was used for the estimation of the percentages of protein secondary structure from circular dichroism data. Several CD curves were generated using the algorithm proposed by [13] to obtain best fit with the experimental data. The CD deconvolution and generation of CD curves were performed using the K2d program at http://www.embl.de/~andrade/k2d/.

### RNA binding assays

RNA binding assays were carried out with 1 pmol ^32^P 5'-labeled oligoribonucleotides. The oligos used were: U_8_C_5_A_8 _(5'UUUUUUUUCCCCCAAAAAAAA3'), C_8_U_5_G_8 _(5'CCCCCCCCUUUUUGGGGGGGG3'), and UUA/C (5'UUAUUAUUCAUUCAUUAUUCA3'). The assays were performed as described previously [14,15], in Tris-HCl pH 8.0, 20 mM KCl, 5 mM MnCl2, 1 mM DTT, 100 ug/ml BSA, 0.8 U Rnasin. Different amounts of trypsin-treated PAB1135 were incubated with the RNA oligos in 20 μl at 37°C for 30 minutes. The samples were resolved on 8% native polyacrylamide gels and visualized on a Phosphorimager (MolecularDynamics).

### Molecular modeling and structural analysis

MODELLER [[16], version 9v1] was used to produce a homology molecular model of PAB1135. The structure of the conserved hypothetical protein Af2318 from *Archaeoglobus fulgidus *(PDB code 2OGK, [17]) was used as a template. This protein shares 40% identity with the PAB1135 sequence, being the top hit in a search through the PDB [18] using BLAST [19]. The parameters used during the modeling exercise were the default of the programs. The alignment used in MODELLER was produced with CLUSTALX [20]. The alignments of the N- and C-terminal regions were cut at the corresponding ends of the crystallographic model.

The homology models were validated with the VERIFY-3D [21] and PROCHECK [22] softwares. The analysis was made through visualization of the superimposed structures using PYMOL [23] and various alignments produced with CLUSTALX. COOT [24] was used for the superposition [25] of the atomic coordinates of the models and PDB files: 2OGK[17], 1JJ2[26], and 1MJI[27]. DALI [28] was also used for analysis of correlated structures.

## Results

### Purification of PAB1135

**Figure 1:**
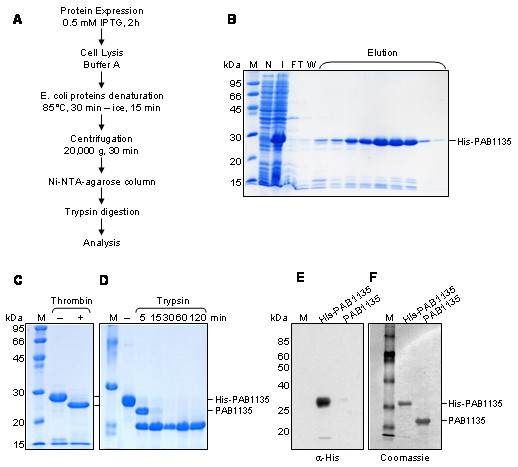
**Purification of PAB1135**. (**A**) Scheme of the protein expression and purification method used. (**B**) Purification of His-PAB1135 by affinity chromatography in Ni-NTA column. (**C**) Cleavage of His-PAB1135 with thrombin for releasing the His-tag. (**D**) Limited proteolysis of His-PAB1135 with trypsin. The His-tag and a few amino acids are released from the N-terminus of PAB1135, originating a very stable protein. Coomassie-stained 15% polyacrylamide gels. M, molecular mass marker; N, extract from non-induced cells; I, induction of His-PAB1135 expression after addition of IPTG; FT, flow through; W, wash; elution with imidazole. (**E**) Immuno blot with anti-His antibody detects only His-PAB1135. (**F**) PVDF membrane stained with coomassie brilliant blue. M, molecular mass marker. PAB1135 was obtained after incubation of His-PAB1135 with trypsin for two hours.

The localization of the *Pyrococcus abyssi *PAB1135 gene in the same operon as the RNase P subunit Rpp30 [7] suggested the involvement of the uncharacterized PAB1135 protein in RNA metabolism. To analyze PAB1135 structure and its association with RNA, PAB1135 gene was cloned and the recombinant protein His-PAB1135 was purified by affinity chromatography. The SDS-PAGE showed protein bands with the expected molecular weight of 21 kDa (Figure 1A,B). The His-tag was released from PAB1135 after cleavage with thrombin (Figure 1C). Limited proteolysis of His-PAB1135 with trypsin resulted in the detection of a very stable protein, corresponding to PAB1135, with a molecular weight close to that expected for the native protein 17.9 kDa (Figure 1D). To ascertain the identity of the peptide released after trypsin cleavage, immunoblot was performed with anti-His antibody and the results show that anti-His is only able to detect the protein before trypsin treatment (Figure 1E,F).

**Figure 2:**
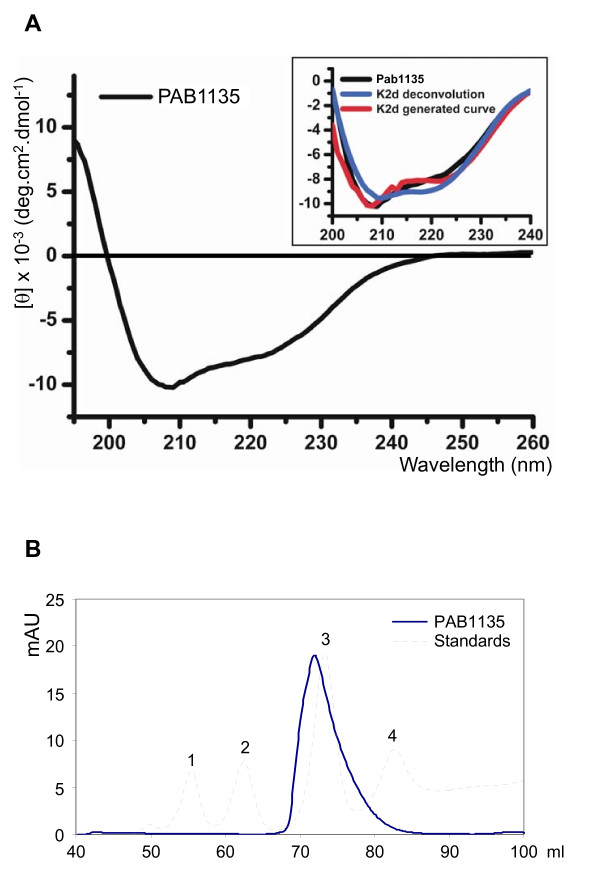
**Structure analysis of PAB1135**. (**A**) Circular dichroism analysis of 2 mg/ml PAB1135. A spectrum with double negative peaks at 208 and 222 nm, and positive peak near 190 nm is detected. The deconvolution of CD spectra (blue line) indicates a >27% and >25% content of alpha and beta structures, respectively. (**B**) Size-exclusion chromatography of PAB1135 after cleavage of His-PAB1135 with trypsin for release of the His-tag. Protein standards used as controls were: 1, albumin, 67 kDa; 2, ovalbumin, 43 kDa; 3, chymotrypsinogen, 25 kDa; 4, ribonuclease A, 13.7 kDa.

Circular dichroism analysis of PAB1135 show a spectrum with double minimum at 208 and 222 nm, and positive peak near 190 nm, as expected for a protein with α/β content, indicating that PAB 1135 has a well defined structure (Figure 2A). The deconvolution of CD spectra using K2d algorithm (Figure 2A; blue line in box) indicates a >27% and >25% content for alpha and beta structures, respectively. A CD curve that better fits the experimental data corresponds to a protein containing 30% and 25% of alpha and beta structures, respectively (Figure 2A; red line in box). These estimations are in accordance with the obtained molecular model for Pa1135 protein.

Interestingly, results from gel filtration assays suggest that PAB1135 forms a homodimer in solution, with an apparent molecular weight of about 30 kDa (Figure 2B), whereas a PAB1135 monomer runs as an approximately 20 kDa protein on SDS-PAGE. This is very similar to the calculated 35.8 kDa molecular weight of a homodimer. In the conditions tested, PAB1135 appears monodisperse in solution since 100% of the mass was accounted for by a single peak at 2.7 nm. The temperature variation between 25°C and 50°C did not affect the results.

**Figure 3:**
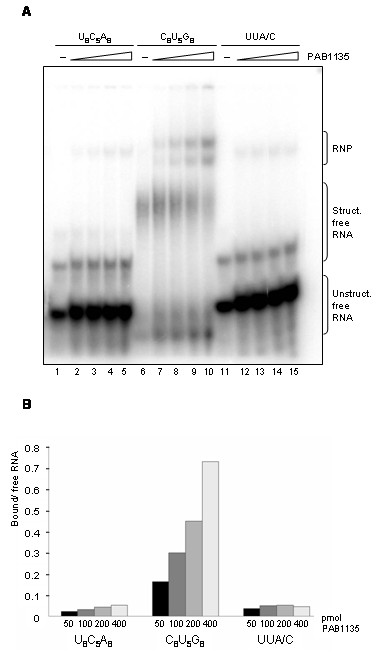
**Analysis of PAB1135 interaction with 21-mer RNA oligonucleotides in vitro**. (**A**) Electrophoretic mobility shift assays with different radiolabeled RNA probes incubated with increasing amounts of purified protein. 50, 100, 200 or 400 pmol of PAB1135 were incubated with 1 pmol of either poly-rU_8_C_5_A_8 _(lanes 2-5), poly-rC_8_U_5_G_8 _(lanes 7-10), or poly-rUUA/C (lanes 12-15) at 37°C for 30 min. RNA-protein complexes were fractionated on 8% native polyacrylamide gels and visualized by phosphorimaging. PAB1135 binds poly-rC_8_U_5_G_8 _efficiently. -, no protein added to the reaction. Free structured and unstructured RNA oligos and PAB1135-RNA complexes are indicated on the right-hand side. (**B**) Quantitation of bands visualized in RNA binding assay. The ratio of protein-bound RNA over free RNA in each lane was calculated for the four different amounts of PAB1135 (50, 100, 200 or 400 pmol).

We had previously analyzed the RNA binding ability of PAB1135 and observed that it does not bind single-stranded RNA oligos, but binds efficiently RNAs that can form low stability hydrogen bonds (J.S. Luz and C.C. Oliveira, unpublished results). Here, we extended these analyses by electrophoresis mobility shift assays. Different amounts of purified PAB1135 were incubated with ^32^P-labeled RNA oligos. Samples were separated by electrophoresis on native polyacrylamide gels and visualized by phosphorimaging (Figure 3). Very weak shifted RNA bands can be visualized when PAB1135 is incubated with the RNA oligos U_8_C_5_A_8 _(low stability secondary structure; ΔG = -1.9 kcal/mol) and UUA/C (single strand) (Figure 3; lanes 1-5 and 11-15, respectively). When incubated with an RNA that forms higher stability secondary structure (oligo C_8_U_5_G_8_, ΔG = -15.9 kcal/mol), however, PAB1135 binds it much more efficiently, and stronger RNA shifted bands can be visualized in the presence of the protein (Figure 3; lanes 6-10). These results indicate that PAB1135 binds RNAs in a non-sequence specific manner, with higher affinity for double-stranded RNAs.

**Figure 4:**
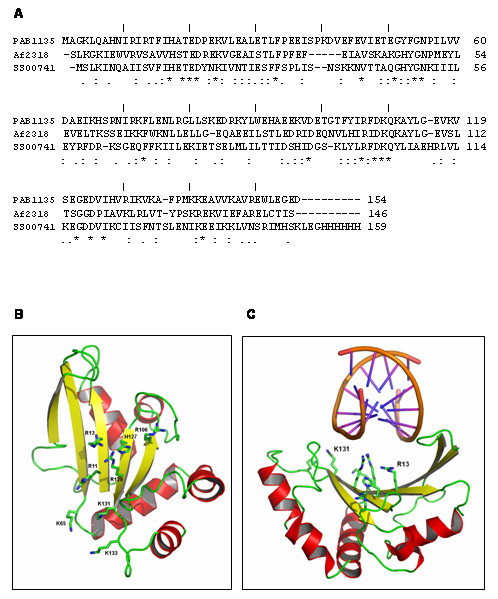
**Sequence alignment of PAB1135**. (**A**) Alignment of PAB1135 from Pyrococcus abyssi with Af2318 from Archaeoglobus fulgidus and SSO0741 from Sulfolobus solfataricus in CLUSTALW. (**B**) Cartoon of the Pa1135 model from MODELLER showing the twisted five-stranded β-sheet with its concave face on one side and α-helices on the other. The side chains of the basic residues on the surface of the β-sheet are shown. (**C**) Cartoon of the model of PAB1135 interacting with RNA.

A BLAST search with the PAB1135 sequence against the PDB shows the *Archaeoglobus fulgidus *2OGK[16] as the closest sequence to PAB1135, with 40% identity and 63% similarity, followed by *Sulfolobus solfataricus *SSO0741 [16] with 27% identity and 48% similarity (Figure 4A). Interestingly, and similar to Pyrococcus abyssi, the gene encoding the Archaeoglobus fulgidus Af2318 protein is present in the same operon as RNase P subunit Rpp30 [7]. Sequence analysis has led to the clustering of these proteins in the UPF0201 family [17]. The structures of members of this protein family show a single α/β domain characterized by a twisted five-stranded anti parallel β-sheet with five α-helices on one side and an unprotected concave surface of the sheet on the other side [17]. A homology model of the PAB1135 based on 2OGK presents good stereochemistry as judged by PROCHECK and VERIFY-3D. The model was used in a DALI search for structural neighbors, yielding a list that was topped by four UPF0201 Archaea structures followed by 1IQ4 and 1JJ2, all with Z score above 7. After these structures the Z score dropped significantly to 3.5 and sequence identity was below 19%. The first three hits were expected and did not yield new information, but the latter two structures are from the ribosomal protein L5 from *Bacillus stearothermophilus *and the large ribosomal subunit from *Haloarcula marismortui*, respectively. In the case of the whole ribosomal subunit, the similarity was also with the L5 protein (Figure 4B).

**Figure 5:**
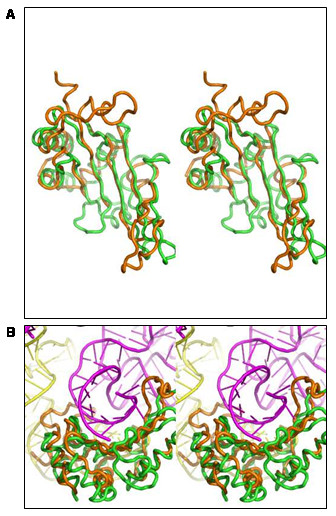
**Superposition of the structures of the PAB1135**, 2OGK, 1JJ2** and **1MJI. (**A**) Superposition of PAB1135 (green) and 1JJ2_D (orange) showing the β-sheet equivalence. (**B**) Protein interacting with the 23S dsRNA (magenta) through its concave surface. The 5S rRNA is shown in yellow.

In light of the functional data showing higher affinity of PAB1135 for double-stranded RNA oligonucleotides, we superposed the structures of the PAB1135, 2OGK, 1JJ2 (only C_α _atoms) and 1MJI. The latter structure, from the L5 ribosomal protein of *Thermus thermophilus*, has the complete protein bound to RNA. The superposition of the PAB1135 model onto the 1JJ2 structure is seen in figure 5. The concave surface of the β-sheet of 1JJ2 is responsible for binding to a double-stranded RNA (dsRNA) region of the 23S rRNA. The size and shape are similar to the surface of PAB1135 although the nature of the residues does not have a clear equivalence (Figure 5). Analysis of the concave surface in the model shows a patch of basic residues formed by Arg11, Arg13, Arg106, His127, Arg129 and Lys131 on the exposed surface of the β-sheet and Lys65 and Lys133 on nearby loops (Figure 5B). Based on the presence of these residues we raised the hypothesis that this region could complex with and stabilize other negatively charged molecules such as the outside of a double-stranded RNA. These residues are not all present in other members of the UPF0201 family which might not bind to RNA. In addition to binding the 23S rRNA, the *Thermus thermophilus *L5 ribosomal protein also interacts with the 5S rRNA through a portion of the protein that is not present in the PAB1135 model and therefore was not considered a good RNA binding site.

## Conclusions

We show that PAB1135 is highly conserved and its structure can be inferred by molecular modeling based on the crystal structures of archaeal UPF0201 proteins. Furthermore, as shown here, PAB1135 binds RNA *in vitro*, with higher affinity for structured RNAs, in accordance with the model's suggestion for the presence of an RNA binding scaffold in the protein. It is possible that PAB1135 binds structured RNAs *in vivo*, such as RNase P RNA and tRNAs.

## Competing interests

The authors declare that they have no competing interests.

## Authors' contributions

JSL purified the recombinant proteins and carried out the activity assays, protein interaction assays and drafted portions of the manuscript. CRRR, stablished the protein purification protocols, helped with the CD analysis, and drafted portions of the manuscript. JARGB coordinated parts of the work, performed the molecular modeling and structural analysis, participated in the interpretation of data and drafted portions of the manuscript. CCO designed, organized and coordinated the experiments, drafted the manuscript and edited the final text. All authors read and approved the final manuscript.
